# Carbapenem-resistant Enterobacterales in Europe: strengthening AMR resilience through coordinated system-level action – lessons from Ukraine

**DOI:** 10.1186/s13756-026-01834-4

**Published:** 2026-10-01

**Authors:** Luise Martin, Arina Zanuzdana, Martin Weber, Nataliia Aliieva, Liliia Mykhajliv, Maryana Svirchuk, Mariia Boiko, Artem Horbachevskyi, Hailie Uren, Brar Christian Piening, Beate Kampmann, Tim Eckmanns, Joachim Seybold, Miriam Songa Stegemann

**Affiliations:** 1 Medical Directorate, Charité – University Medical Center Berlin, Corporate Member of Freie Universität Berlin and Humboldt-Universität zu Berlin, Berlin, Germany; 2 Department of Pediatric Respiratory Medicine, Immunology and Critical Care Medicine, Charité – University Medical Center Berlin, Corporate Member of Freie Universität Berlin and Humboldt-Universität zu Berlin, Berlin, Germany; 3 Department of Infectious Disease Epidemiology, Robert Koch Institute, Berlin, Germany; 4 First Lviv Territorial Medical Union, Lviv, Ukraine; 5 Danylo Halytsky Lviv National Medical University, Lviv, Ukraine; 6 Regional Clinical Hospital of Ivano-Frankivsk, Ivano-Frankivsk, Ukraine; 7 Public Health Center of the Ministry of Health of Ukraine, Kyiv, Ukraine; 8 Griffith University, Gold Coast, Australia; 9 Institute of Hygiene and Environmental Medicine, National Reference Centre for Surveillance of Nosocomial Infections, Charité – University Medical Center Berlin, Corporate Member of Freie Universität Berlin and Humboldt-Universität zu Berlin, Berlin, Germany; 10 Charité Centre for Global Health, Charité – University Medical Center Berlin, Corporate Member of Freie Universität Berlin and Humboldt-Universität zu Berlin, Berlin, Germany; 11 Department of Infectious Diseases, Respiratory and Critical Care Medicine, Campus Virchow-Klinikum and Campus Charité Mitte, Charité – University Medical Center Berlin, Corporate Member of Freie Universität Berlin and Humboldt-Universität zu Berlin, Berlin, Germany

**Keywords:** Carbapenem-resistant Enterobacterales, Antimicrobial resistance, Europe, Ukraine, Infection prevention and control, Antimicrobial stewardship, Implementation, Health security

## Abstract

Carbapenem-resistant Enterobacterales (CRE) have emerged as one of the most serious antimicrobial resistance (AMR) threats in Europe. Rising incidence, substantial mortality, and marked heterogeneity across the EU/EEA pose a growing challenge. In Ukraine, an already substantial AMR burden and pre-existing limitations in diagnostic and infection prevention capacity, infectious diseases and antimicrobial stewardship (AMS) expertise, workforce resources, and access to effective antimicrobials have been further exacerbated by the war, including through damage to healthcare infrastructure and disruption of supply chains. These conditions facilitate the emergence and spread of CRE. As patient movement across Europe continues, CRE in Ukraine has become a shared European health security challenge. Although numerous national and international initiatives support AMR control in Ukraine, their impact is limited by fragmented implementation, insufficient coordination, and limited visibility across stakeholders. Drawing on hospital partnerships, multidisciplinary expert discussions, and emerging national coordination efforts in Ukraine, we argue that sustainable CRE control requires an integrated, system-level framework combining strengthened microbiological diagnostics and surveillance, infection prevention and control, infectious diseases and AMS expertise, and equitable access to effective antimicrobials. These components should be embedded within a coordinated national framework that aligns international support with national priorities. We describe the recently established, Ukrainian-led Public Health Center of the Ministry of Health of Ukraine (PHC) AMR Coordination Working Group as an emerging example, combining regular technical engagement, structured stakeholder collaboration, and plans to map international AMR-related activities in Ukraine. This approach may inform future European efforts to organise international AMR support in other high-burden and crisis-affected settings.

Carbapenem-resistant Enterobacterales (CRE) have become a major infectious disease challenge confronting European health systems [1, 2]. Across the EU/EEA, bloodstream infections due to carbapenem-resistant *Klebsiella pneumoniae* have risen sharply since 2019, reversing previous progress and moving the EU further away from its reduction targets [3, 4]. Despite this overall trend, carbapenem resistance remains highly heterogeneous across Europe, exceeding 50% in several countries while remaining below 1% in others [3]. CRE infections are associated with substantial attributable mortality, prolonged hospital stays, and considerable economic burden [5, 6].

The marked heterogeneity in CRE epidemiology reflects differences in health system preparedness, including microbiological diagnostic capacity, IPC infrastructure, specialist infectious diseases and AMS expertise, workforce resources, and access to effective antimicrobials. At the same time, CRE dissemination is facilitated by high-risk clonal lineages and plasmid-mediated gene transfer; in *K. pneumoniae*, the convergence of resistance and virulence traits represents an additional concern [7–9]. Consequently, while national epidemiology differs, CRE remains a shared European health security challenge in a region characterised by extensive patient mobility.

Ukraine illustrates these system pressures in an especially acute form. Clinicians in many Ukrainian hospitals and other healthcare facilities confront CRE under some of the most challenging conditions in contemporary healthcare: damaged infrastructure, insufficient laboratory capacity, severe workforce shortages, supply-chain disruptions, and the ongoing demands of war [10–15].

The epidemiological burden reflects these conditions. In Ukrainian military hospitals, carbapenem resistance among *Klebsiella pneumoniae* wound isolates increased from 32.8% during 2014–2020 to 90.6% for meropenem and 81.2% for imipenem during 2022–2023 [14]. During the conflict, infections with carbapenem-resistant organisms accounted for up to 60% of healthcare-associated infections in Ukrainian hospitals [15]. A study of 56 *K. pneumoniae* wound isolates recovered from Ukrainian soldiers between 2022 and 2023 further illustrated the severity of the resistance burden: all isolates were multidrug-resistant, 78.6% were extensively drug-resistant, and many harboured both carbapenemase genes and virulence determinants, highlighting the convergence of resistance and virulence [16]. Notably, wounds in Ukrainian soldiers were predominantly colonised with bacteria with limited antimicrobial resistance before their first contact with the healthcare system, suggesting that acquisition of resistant organisms may occur during subsequent medical care [17]. Moreover, national surveillance data showed a 7-8-fold increase in the proportion of typical nosocomial pathogens, including *K. pneumoniae* and *P. aeruginosa*, in wounds during prolonged treatment [18].

Patients often require urgent, complex medical care, yet essential components of infection management - timely microbiological diagnostics, IPC capacity, infectious diseases and AMS expertise - are limited or unavailable [12, 19]. Limited access to effective antimicrobials further constrains treatment options. Beyond cost, several newer CRE-active agents lack formal registration in Ukraine, while wartime conditions have weakened supply chains and distribution to frontline hospitals [10]. Consequently, life-saving therapies may remain inaccessible, underscoring the need for equitable access mechanisms and reliable procurement. Access to newer reserve antibiotics must, however, be accompanied by quality microbiological diagnostics, ID and AMS expertise to ensure appropriate patient selection, effective use, and preservation of their effectiveness [20].

The war in Ukraine has amplified and regionalised an already serious threat by creating structural conditions that facilitate the emergence, transmission, and cross-border spread of CRE through the interaction of conflict, patient mobility, and uneven health system preparedness [21]. Together, these factors position CRE not only as an infectious diseases challenge but also as a European health security issue. Europe’s ability to contain CRE therefore depends on the resilience of all its health systems, including Ukraine´s. Vulnerabilities in one country inevitably increase regional risk by facilitating the amplification and dissemination of highly resistant pathogens that are difficult to detect, trace, control, and treat. This reflects the fundamentally interconnected nature of antimicrobial threats in a highly mobile Europe. Strengthening microbiological diagnostics, IPC, infectious diseases and AMS expertise, and equitable antimicrobial access across the region is therefore essential [22–25].

Experience from the ongoing hospital partnership between Charité – Universitätsmedizin Berlin and hospitals in Lviv and Ivano-Frankivsk has highlighted both the value and the limitations of bilateral collaboration. While regular case discussions, shared infectious diseases and AMS protocols, IPC mentoring, and bidirectional learning supported clinical decision-making, many of the barriers encountered, including limited diagnostic capacity, workforce shortages, limited surveillance capacity, and access to newer CRE-active antimicrobials, extend beyond the scope of individual hospital partnerships and require coordinated action at national and international levels. These practical experiences informed subsequent discussions at the multidisciplinary CRE Symposium in Berlin and the ESCMID Supports Ukraine Meeting in Munich.

The multidisciplinary CRE Symposium held in Berlin in November 2025, convened by Charité – Universitätsmedizin Berlin, the Robert Koch Institute, and Lviv First Medical Union, brought together clinicians, public health experts, and international partners to discuss the growing regional implications of CRE. Clinicians from Germany described rising but comparatively contained CRE incidence, emphasising preparedness, coordinated national strategies, digital surveillance, and integrated genomic approaches to detect emerging transmission. In contrast, experts from Ukraine highlighted that effective CRE control depends not only on IPC and AMS, but also on sustained investment in diagnostics, workforce capacity, education, and equitable access to effective antimicrobials. The discussions underscored that many of the barriers identified in Ukraine were system-level challenges requiring action beyond individual institutions and bilateral partnerships.

These conclusions were reinforced during the ESCMID Supports Ukraine Meeting in April 2026, a side event at ESCMID Global 2026 in Munich co-organised by the Robert Koch Institute, Charité – Universitätsmedizin Berlin, and the US Centers for Disease Control and Prevention, with support from the ESCMID leadership. The meeting brought together international stakeholders and partners engaged in AMR activities in Ukraine and pointed to the need for closer alignment of external support with national priorities.

Ukraine has demonstrated a strong commitment to tackling AMR, with initiatives led by national authorities, academic institutions, professional societies, humanitarian and international organisations, and technical partners. Within this broader landscape, the Public Health Center of the Ministry of Health of Ukraine (PHC), as the national public health institution responsible for coordinating AMR activities, plays a central role in convening national and international stakeholders.

The PHC has recently established an AMR Coordination Working Group with technical support from the Robert Koch Institute and Charité – Universitätsmedizin Berlin. The initiative combines regular technical meetings, stakeholder consultations, and plans for a digital dashboard to map AMR-related activities of international stakeholders in Ukraine, identify synergies, and support implementation of Ukraine’s National Action Plan on AMR.

Although still at an early stage, the PHC-led Working Group may provide an emerging governance model for integrating technical interventions and projects within a structured national coordination framework while aiming to provide a comprehensive overview of current activities at national, regional and local levels. Drawing on lessons from the Charité - Ukraine hospital partnerships, subsequent multidisciplinary discussions, and this developing coordination model, we propose a general implementation framework for CRE control that combines established technical priorities with an enabling national coordination mechanism.

## Towards an integrated implementation framework for CRE control

### Core technical components

Effective CRE control depends on five interdependent technical components: microbiological diagnostics, IPC, infectious diseases and AMS expertise, and equitable access to effective antimicrobials.

Timely and reliable microbiological diagnostics are fundamental to CRE control. Early identification of colonised and infected patients enables timely IPC measures, including isolation and cohorting, to interrupt transmission. Pathogen-directed treatment can reduce reliance on broad-spectrum antibiotics and mitigate selective pressure. Reliable identification and susceptibility data underpin effective infectious disease consultation, rational antimicrobial use, and surveillance of emerging resistance.

Given the limited therapeutic options for CRE, prevention through effective IPC is foundational to any control strategy. Strengthening IPC capacity, including surveillance for early detection of transmission, isolation practices, environmental hygiene, and adequate staffing, is essential to interrupt transmission, particularly in high-burden settings. Infectious diseases expertise and robust AMS programmes are central to clinical decision-making, ensuring appropriate management and rational antimicrobial use. Equitable access to effective antimicrobials must be embedded within these systems to ensure targeted use and limit further resistance.

### Enabling coordination mechanism

While these technical priorities are individually well established and anchored in the National Action Plan, their successful implementation depends on effective national coordination. Without coordination, technical interventions funded and supported by international partners risk remaining fragmented and insufficiently aligned with national priorities.

The PHC-led AMR Coordination Working Group provides an emerging mechanism to coordinate national and international AMR activities, align partner investments with Ukraine´s National Action Plan on AMR and national legislation, improve transparency, reduce duplication, and strengthen accountability. Key implementation mechanisms include:

- Regular technical coordination meetings;
- Stakeholder consultations bringing together national authorities and international partners, including WHO headquarters, WHO regional and country offices, ECDC and the US CDC;
- A digital workspace and plans for a digital dashboard to provide an up-to-date overview of AMR-related activities of international stakeholders in Ukraine and support implementation of the National Action Plan.

CRE is already reshaping medical practice, straining hospital systems, and compromising treatment outcomes. Yet it remains a containable threat, provided Europe moves beyond fragmented initiatives towards coordinated implementation. Sustainable CRE prevention and control require microbiological diagnostics, IPC, infectious diseases and AMS expertise, and equitable access to effective antimicrobials to be integrated within a nationally led implementation framework. The recently established PHC-led AMR Coordination Working Group represents an important initial step towards this goal by helping to align international support, reduce duplication, and strengthen long-term sustainability. Lessons from Ukraine may inform similar implementation frameworks for coordinated CRE control in other high-burden and crisis-affected settings.

## Data Availability

No datasets were generated or analysed during the current study.

## Declarations

### AI use

During the preparation of this work the authors used ChatGPT in order to support language editing and improve clarity of English. After using this tool, the authors reviewed and edited the content as needed and take full responsibility for the content of the published article.

## Abbreviations

CRE: Carbapenem-resistant Enterobacterales
AMR: Antimicrobial resistance
IPC: Infection prevention and control
AMS: Antimicrobial stewardship
ID: Infectious diseases
EU/EEA: European Union/European Economic Area
PHC: Public Health Center of the Ministry of Health of Ukraine

## References

[1] European Centre for Disease Prevention and Control. Carbapenem-resistant Enterobacterales – third update. Stockholm: European Centre for Disease Prevention and Control; 2025.

[2] European Commission HSC. Opinion on the rapidly increasing incidence of carbapenem-resistant Enterobacterales (CRE) in healthcare settings. Brussels: European Commission; 2025.

[3] European Centre for Disease Prevention and Control. Country summaries – antimicrobial resistance in the EU/EEA 2023. Stockholm: European Centre for Disease Prevention and Control; 2024.

[4] Cooke E, Rendi-Wagner P. Rising antimicrobial resistance in Europe: EMA and ECDC outline urgent actions to achieve EU targets. Lancet Reg Health Eur. 2025;59:101539. 10.1016/j.lanepe.2025.101539 PMC12744718 41466606

[5] Kingston R, Vella V, Pouwels KB, Schmidt JE, Abdelatif El-Abasiri RA, Reyna-Villasmil E, et al. Excess resource use and cost of drug-resistant infections for six key pathogens in Europe: a systematic review and Bayesian meta-analysis. Clin Microbiol Infect. 2024;30(Suppl 1):S26–36. 10.1016/j.cmi.2023.12.013 38128781

[6] Paniagua-García M, Bravo-Ferrer JM, Pérez-Galera S, Kostyanev T, de Kraker MEA, Feifel J, et al. Attributable mortality of infections caused by carbapenem-resistant Enterobacterales: results from a prospective, multinational case-control-control matched cohorts study (EURECA). Clin Microbiol Infect. 2024;30(2):223–30. 10.1016/j.cmi.2023.11.008 38267096

[7] Fröding I, David S, Yeats C, Abu-Dahab K, Albiger B, Alikhan NF, et al. Persistent spread of carbapenemase-producing Klebsiella pneumoniae in acute care hospitals in 36 European countries (the CCRE survey): a prospective, multicentre, cross-sectional, epidemiological, microbiological, and genomic surveillance study. Lancet Microbe. 2026;7(6):101320. 10.1016/j.lanmic.2025.101320 PMC13259977 42127954

[8] Gomez-Simmonds A, Annavajhala MK, Seeram D, Hokunson TW, Park H, Uhlemann AC. Genomic epidemiology of carbapenem-resistant Enterobacterales at a New York City hospital over a 10-year period reveals complex plasmid-clone dynamics and evidence for frequent horizontal transfer of bla (KPC). Genome Res. 2024;34(11):1895–907. 10.1101/gr.279355.124 PMC11610580 39366703

[9] Yang X, Dong N, Chan EW, Zhang R, Chen S. Carbapenem Resistance-Encoding and Virulence-Encoding Conjugative Plasmids in Klebsiella pneumoniae. Trends Microbiol. 2021;29(1):65–83. 10.1016/j.tim.2020.04.012 32448764

[10] Uren HD, Aliieva N, Matolinets N, Berrey BH, Holcomb JB, Voitovych O, et al. Conflict zone medical evacuations catalyzing antimicrobial resistance spread and threatening regional health: A retrospective comparative observational study. J Trauma Acute Care Surg. 2025;99(3S Suppl 1):S45–54. 10.1097/TA.0000000000004729 40768657

[11] Zelinskyy G, Koval T, Schrammel U, Elsner C, Ackermann J, Marchenko O, et al. Establishing an EU-compliant diagnostic facility for infectious diseases under war conditions in Poltava, Ukraine. Front Public Health. 2026;14:1840777. 10.3389/fpubh.2026.1840777 PMC13260340 42293605

[12] Haque U, Bukhari MH, Fiedler N, Wang S, Korzh O, Espinoza J, et al. A Comparison of Ukrainian Hospital Services and Functions Before and During the Russia-Ukraine War. JAMA Health Forum. 2024;5(5):e240901. 10.1001/jamahealthforum.2024.0901 PMC11102023 38758566

[13] Vodianyk A, Diomin E, Husakov A, Havrilov I, Horbachevskyi A, Habicht J. Healthcare-associated infections and antimicrobial use in Ukrainian acute care hospitals involved in treatment of casualties of war: multi-centre cross-sectional study in 2024. J Hosp Infect. 2025;162:333–8. 10.1016/j.jhin.2025.05.015 PMC12351372 40484243

[14] Kovalchuk V, Kondratiuk V, McGann P, Jones BT, Fomina N, Nazarchuk O, et al. Temporal evolution of bacterial species and their antimicrobial resistance characteristics in wound infections of war-related injuries in Ukraine from 2014 to 2023. J Hosp Infect. 2024;152:99–104. 10.1016/j.jhin.2024.06.011 38997008

[15] Molina KC, Bills C, Bebarta VS, Fishchuk R, Mueller SW, Peers JL, et al. The evolving frontline of antimicrobial resistance: adapting testing and treatment for austere and combat settings. Mil Med. 2026. 10.1093/milmed/usag024 41816933

[16] Kondratiuk V, McGann P, Jones BT, Fomina N, Nazarchuk O, Kovalchuk V. Antibiotic resistance characteristics and genetic determinants of hypervirulence in Klebsiella pneumoniae isolated from wounded individuals in Ukraine. J Hosp Infect. 2026;167:232–7. 10.1016/j.jhin.2025.10.016 41177264

[17] Dumchev K, Mc Gann P, Danyliuk O, Metreveli M, Musich TA, Bennett JW. Microbial Flora in War Wounds from the Ukrainian Front Line. N Engl J Med. 2026;394(9):926–8. 10.1056/NEJMc2512101 41740038

[18] Public Health Center of the Ministry of Health of Ukraine. Report on the Results of Enhanced Epidemiological Surveillance of Antimicrobial Resistance among Microorganisms Causing Purulent Wound Infections in Individuals Wounded as a Result of Military Hostilities, 2023–2024. Kyiv, Ukraine: Public Health Center of the Ministry of Health of Ukraine; 2025.

[19] Spiegel P, Martinez E, Abrahim O, Undie CC, Altare C, Schmid B, et al. Johns Hopkins Center for Humanitarian Health-Lancet Commission on health, conflict, and forced displacement: health in a world of crises and impunity. Lancet. 2026;407(10543):2065–140. 10.1016/S0140-6736(26)00564-7 42155503

[20] Patel TS, Sati H, Lessa FC, Patel PK, Srinivasan A, Hicks LA, et al. Defining access without excess: expanding appropriate use of antibiotics targeting multidrug-resistant organisms. Lancet Microbe. 2024;5(1):e93–8. 10.1016/S2666-5247(23)00256-2 PMC10789610 37837986

[21] Sandfort M, Hans JB, Fischer MA, Reichert F, Cremanns M, Eisfeld J, et al. Increase in NDM-1 and NDM-1/OXA-48-producing Klebsiella pneumoniae in Germany associated with the war in Ukraine, 2022. Euro Surveill. 2022;27(50). 10.2807/1560-7917.ES.2022.27.50.2200926 PMC9808319 36695468

[22] Davido B, Ny S, van Lingen C, Årdal C, Alonso Irujo L, Linnros S, et al. Strengthening antimicrobial resistance governance in Europe: a coordinated one health approach. Lancet Reg Health Eur. 2026;61:101540. 10.1016/j.lanepe.2025.101540 PMC12767864 41498071

[23] Maraolo AE, Ong DSY, Cimen C, Howard P, Kofteridis DP, Schouten J, et al. Organization and training at national level of antimicrobial stewardship and infection control activities in Europe: an ESCMID cross-sectional survey. Eur J Clin Microbiol Infect Dis. 2019;38(11):2061–8. 10.1007/s10096-019-03648-2 PMC6800847 31396830

[24] Uren HD, Aliieva N, Jensen MPN, Matolinets N, Holcomb JB, Fedchyshyn N, et al. Can antimicrobial stewardship programmes be implemented during an armed conflict? A retrospective interventional cohort study. J Antimicrob Chemother. 2026;81(6). 10.1093/jac/dkag115 42153395

[25] Granata G, Petersen E, Capone A, Donati D, Andriolo B, Gross M, et al. The impact of armed conflict on the development and global spread of antibiotic resistance: a systematic review. Clin Microbiol Infect. 2024;30(7):858–65. 10.1016/j.cmi.2024.03.029 38556213

